# Investigating the Characteristics of the Laser Powder Bed Fusion of SiCp/AlSi10Mg Composites: From a Single Track to a Cubic Block

**DOI:** 10.3390/mi16060697

**Published:** 2025-06-11

**Authors:** Ying He, Gang Xue, Haifeng Xiao, Haihong Zhu

**Affiliations:** 1 National Key Laboratory of Particle Transport and Separation Technology, Tianjin 300180, China; heying_csu@163.com; 2 Wuhan National Laboratory for Optoelectronics, Huazhong University of Science and Technology, Wuhan 430074, China; 3 Wuhan Marine Machinery Plant Co., Ltd., Wuhan 430085, China

**Keywords:** laser powder bed fusion, SiCp/AlSi10Mg composites, characteristics, single track, mechanical properties, wear

## Abstract

Laser powder bed fusion (LPBF) of SiCp/AlSi10Mg is promising in many industrial fields. In this paper, the characteristics of a 15 wt.% 1200 mesh SiCp/AlSi10Mg metal matrix composite fabricated by LPBF were investigated systematically, i.e., from a single track to a block. It was found that when the laser energy input was high enough, the single track was continuous and not distorted; when the laser energy input was low, the single track was unstable and wrinkled. The densification of the LPBFed composite sample was influenced significantly by the surface morphologies and geometric dimensions of the single tracks. As high as 98.9% relative density was achieved when the optimized processing parameters were used. Because of the good wettability and the interfacial reaction during the process, the interface of SiC and the matrix showed good bonding. Near the interface of SiC and the matrix, needle-shaped phase Al_4_SiC_4_ could be found both in the single track and block, and the faceted particle Si was formed in the block because of the interfacial reaction. The microhardness of the LPBFed SiCp/AlSi10Mg composites was much higher than that of the LPBFed unreinforced AlSi10Mg. A coefficient of friction of 0.178 and wear rate of 2.02 × 10^−4^ mm^3^/(N⋅m) were achieved for the LPBFed composites. The main wear mechanism was delamination wear, accompanied by abrasive wear. The maximum yield strength and ultimate compressive strength were 566.6 MPa and 764.1 MPa, respectively. The fracture mode of the LPBFed composites is mainly brittle fracture. This study provides a theoretical and technical basis for LPBFed SiCp/AlSi10Mg 3D parts.

## 1. Introduction

Because of its high thermal conductivity, specific stiffness, wear resistance, and low density, the SiC particle-reinforced Al matrix composite is a popular metal matrix composite (MMC) in industry [1,2], such as electronic circuits, aerospace, and so on. With the increasing demand for light weight materials, the configuration of components is becoming more and more complex. Traditional techniques, such as casting, forging, and powder metallurgy, are difficult to use in fabricating them. Laser powder bed fusion (LPBF), one additive manufacturing (AM) technique, selectively melts/solidifies the desired powder area guided by a 3D CAD file. Compared with the traditional processes, this technique has the advantages of freedom in design and the ability to easily fabricate 3D parts with a complex configuration, high accuracy, and high performance, even for multi-metal scaffolds with nanoarchitectures [3,4,5].

LPBF is promising for use in fabricating high-performance SiCp/AlSi10Mg composite parts with complex configurations. In recent years, there have been some studies on the LPBFed SiCp/AlSi10Mg composites. Famodimu et al. [6] investigated the influence of scanning speed on the characteristics of single tracks and blocks with 5 vol.% and 10 vol.% SiC. They found that instability and distorted single tracks formed when the scanning speed was too high. However, cracks also formed when the scanning speed was too low. In Gu et al.’s studies, 96% relative density, 214 HV_0.1_ microhardness, a 0.39 coefficient of friction (COF), and a 1.56 × 10^−5^ mm^3^/(N·m) wear rate were achieved when the SiC content was 20 wt.% [7]. Furthermore, they disclosed that Al_4_SiC_4_ strips with micron-level sizes were formed due to the in situ reaction between SiC particles and the Al matrix during the LPBF process; therefore, the Young’s modulus and hardness of the LPBFed AlSi10Mg/SiC composites were higher than those of LPBFed AlSi10Mg. However, SiC particles in the composite were more effective because of their higher strength and Young’s modulus than those of Al_4_SiC_4_ [8]. Ghosh et al. [9] studied the wear performance and mechanism of LPBFed SiCp/AlSi10Mg. It was found that the specific wear rate increased as the SiC particle size decreased. As the SiC content increased, the wear resistance of the composite initially increased and then remained stable after the SiC content exceeded 20 vol.%. The wear mechanisms were mainly sliding and abrasive. Yanase et al. obtained a compressive stress of more than 600 MPa for LPBFed SiCp/ AlSi10Mg at an optimum energy density [10]. Chen et al. [11] investigated the microstructure, defects, thermal expansion coefficient, and mechanical properties of LPBFed with 10 vol.% SiCp/AlSi10Mg. In the end, a 351 MPa ultimate tensile strength, a 861.9 MPa compressive strength, a 121.9 GPa compressive modulus, and a 18.23 × 10^−6^/°C thermal expansion coefficient were achieved.

In order to increase the relative density and performance of LPBFed SiCp/AlSi10Mg, Miyauchi et al. [12] utilized a remelting scan, and Ji et al. [8] utilized a near-spherical β-SiC powder. Our previous research [13] also obtained 97.7% relative density and 341.9 MPa tensile strength by optimizing the processing parameters. In this study, 300 mesh 15 wt.% SiC was used, and only block samples were investigated. Furthermore, we investigated the effects of the SiC particle size on the properties. It was found that the relative density, microhardness, and compressive strength increased as the SiC particle size decreased [14]. Therefore, using a small SiC particle size is beneficial for LPBFed SiCp/AlSi10Mg. However, the relative density and performance of LPBFed SiCp/AlSi10Mg composites still require further improvement, and the defects of LPBFed SiCp/AlSi10Mg composites also need to be further reduced. Moreover, the mechanism for decreasing the defects of LPBF needs to be investigated. LPBF is a layer-by-layer technique. All the layers are composed of single tracks. Thus, the geometric topography and dimensions of the single track directly determine the bonding of the present track and layer with the previous track and layer [15]. Simultaneously, the shape and surface morphology of the molten pool influence the density and microstructure of the block sample. Furthermore, systematic studies of the evolution from a single track to a cubic block of LPBFed SiCp/AlSi10Mg composites were very limited until now. This paper investigated the effects of the processing parameters on the surface morphology and molten pool characteristics of the single tracks. Furthermore, the characteristics, e.g., relative density, microstructure, microhardness, tribological property, and compressive strength, of LPBFed SiCp/AlSi10Mg block samples were also investigated in this paper. In order to obtain good performance, a SiC power as fine as 1200 mesh was used. The content SiC was fixed at 15 wt.%.

## 2. Materials and Methods

**Figure 1:**
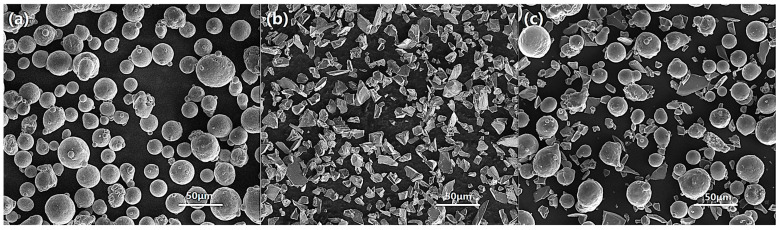
SEM micrographs of the starting powder: (**a**) AlSi10Mg; (**b**) SiC; (**c**) mixture.

Spherical shape AlSi10Mg powders (Figure 1a) and irregular shape SiC powders (Figure 1b) were utilized in this experiment. The average particle sizes of AlSi10Mg powders and SiC powders were 33.7 μm (−300 mesh) and 10.8 μm (−1200 mesh), respectively. The suppliers of AlSi10Mg powders and SiC powders were Hunan Hengji Powder Technology Co., Ltd., Yueyang, China, and Beijing Guanjinli New Material Technology Co., Ltd., Beijing, China, respectively. Before the LPBF process, two powders were blended in a tumbling mixer with a weight ratio of 85:15. The rotation rate of the mix process was 80 rpm and the time of rotation was 2 h with a ball-to-powder weight ratio of 2:1. As shown in Figure 1c, the shapes of the mixture powders do not change, and the distribution of SiC particles in the mixture are almost homogeneous.

**Figure 2:**
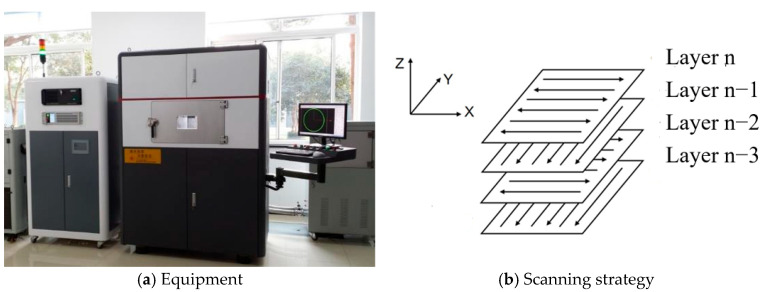
LPBF equipment (**a**) and diagram of scanning strategy (**b**).

All the LPBF experiments were performed on a self-developed LPBF machine (Figure 2a). During the process, the atmosphere was controlled, with an O_2_ concentration below 100 ppm by flowing Ar. A continuous wave fiber laser (Model YLR-500-WC-Y14, IPG (Beijing) Fiber Laser Technology Co., Ltd., Beijing, China), with a center wavelength of 1070 nm and maximum output power of 500 W, was equipped on the machine. The M^2^ was less than 1.1. The forming area of the machine was 250 mm × 250 mm × 300 mm. The laser spot size was focused at about 120 μm. The single track length was 15 mm. When block samples were fabricated, an alternating x/y raster strategy with a 90° phase angle was used (Figure 2b). The size of the cubical samples was 15 mm × 15 mm × 12 mm. Other processing parameters are listed as shown in Table 1.

The relative density $\rho_{RD}\;$of the LPBFed composites sample was calculated as Equation (1):

(1) $$\rho_{RD}=\frac{\rho }{\rho_{TD}}100\%$$

The density of the LPBFed composite sample ρ was measured by the Archimedes Principle. The measurements were repeated 3 times and the value was their average. Theoretical density $\rho_{TD}$ of the composites was calculated by Equation (2):

(2) $$\rho_{TD}=\rho_{AlSi10Mg}\cdot V_{AlSi10Mg}+\rho_{SiC}\cdot V_{SiC}$$

where $\rho_{AlSi10Mg}$ is the theoretical density of AlSi10Mg, $\rho_{SiC}$ is the theoretical density of SiC, and, here, $\rho_{AlSi10Mg}$= 2.68 g/cm^3^ and $\rho_{SiC}=$3.21 g/cm^3^. $V_{AlSi10Mg}$ and $V_{SiC}$ are the volume fractions of AlSi10Mg and SiC, respectively.

Confocal laser scanning microscope (CLSM, Keyence VK-X200, Keyence Corporation, Osaka, Japan) was used to detect the surface morphology. The metallograph samples were grounded and polished according to the standard metallographic examination procedures fabricated for the cubic sample. After that, etching was carried out by a solution ratio of water, HNO_3_, HCl and HF of 95:2.5:1.5:1.0. Field-emission scanning electron microscopy (FE-SEM, FEI Nova 400 Nano, FEI Company, Hillsboro, OR, USA) and optical microscopy (AE2000, Motic Instruments Inc., Kowloon Bay, Hong Kong, China) were used to watch the molten pool and microstructure. The distribution of the element was measured by using EDSanalysis using the SEM images. Microhardness was measured by the DHV-1000Z microhardness tester using a load of 200 g and a dwell time of 20 s. Each datum was the average of twenty data obtained from testing of different locations. The compressive samples were machined according to the ASTM E9-89a (2000) [16] standard with a size of Φ10 mm × 15 mm. The compressive test was performed using an AG-100kN model universal material-testing machine at a tensile rate of 1 mm/min and room temperature. Each final value was the average of the three repeated t. After rupture, FE-SEM was used to watch the fracture morphology.

A ball-on-disk testing machine (Bruker UMT TriboLab, Bruker Corporation, Billerica, MA, USA) was used to test the tribological property. During the wear test, no lubrication was used and the testing was performed at ambient temperature. Before the measurement, the sample was polished up to 1200 grit SiC papers. 

Si_3_N_4_ balls with diameter of 6.35 mm were loaded against the sample. Reciprocating motion was utilized as the moving mode of the ball. The sliding speed was kept constant of 10 mm/s. The total test time was 3600 s. The coefficient of friction (COF) was recorded automatically during the test. The wear rate δ was determined as Equation (3):

(3) $$\delta =\frac{M_{b}-M_{f}}{\rho *\Gamma *d}$$

where $M_{b}$ and $M_{f}\;$ are the weight of the sample before and after wear test, respectively. The weights were measured by using an electronic balance, the accuracy of which was 10^−2^ mg. Before the test, the sample was cleaned using ethanol and dried. *Γ* is the applied normal load and d is the total sliding distance. In this experiment, Γ=10 N and d=10 mm. The worn surface was also observed using FE-SEM.

## 3. Results and Discussion

### 3.1. Single Track

**Figure 3:**
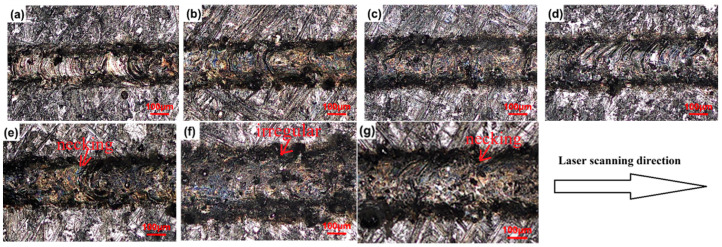
Typical surface morphologies of the LPBFed single tracks with a fixed scanning speed of 300 mm/s and different laser powers of (**a**) 300 W, (**b**) 340 W, (**c**) 370 W, (**d**) 400 W, (**e**) 430 W, (**f**) 460 W, and (**g**) 500 W.

**Figure 4:**
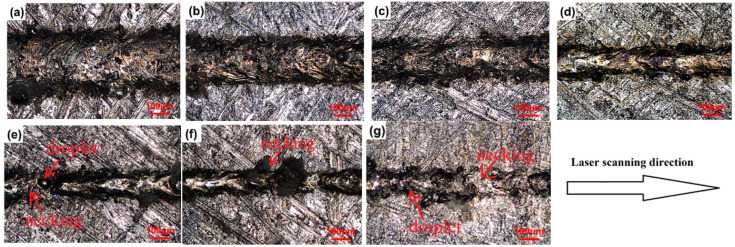
Typical surface morphologies of the LPBFed single tracks with a fixed laser power of 500 W and different scanning speeds of (**a**) 300 mm/s, (**b**) 600 mm/s, (**c**) 900 mm/s, (**d**) 1200 mm/s, (**e**) 1500 mm/s, (**f**) 1800 mm/s, and (**g**) 2100 mm/s.

The surface morphologies of the LPBFed single tracks fabricated by different laser powers and a constant scanning speed of 300 mm/s are shown in Figure 3. The surface morphologies of single tracks of LPBFed SiCp/AlSi10Mg composites fabricated at different scanning speeds and a constant laser power of 500 W are shown in Figure 4. It can be seen from the figures that laser power and scanning speed both have significant influences on the surface morphology of the single track. When the laser power decreases or the scanning speed increases, the surface morphologies of the single tracks become distorted and irregular. Irregular and discontinuous tracks, e.g., neckings and droplets, appear when the scanning speed exceeds 1500 mm/s with laser power kept constant at 500 W, as shown in Figure 4e,f and below 340 W when the scanning speed is 300 mm/s, as shown in Figure 3a,b.

**Figure 5:**
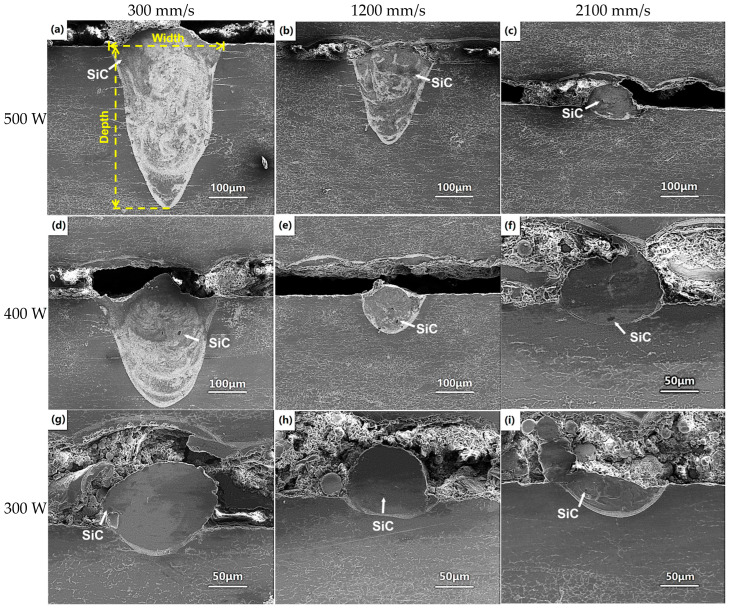
The typical molten pools of the single tracks with different laser powers and scanning speeds: (**a**) 500 W, 300 mm/s; (**b**) 500 W, 1200 mm/s; (**c**) 500 W, 2100 mm/s; (**d**) 400 W, 300 mm/s; (**e**) 400 W, 1200 mm/s; (**f**) 400 W, 2100 mm/s; (**g**) 300 W, 300 mm/s; (**h**) 300 W, 1200 mm/s; and (**i**) 300 W, 2100 mm/s.

Figure 5 shows the typical molten pools of the single tracks when different laser powers and scanning speeds are utilized. The molten pool depths shown in Figure 5a,b,d are very large when the scanning speed is below 1200 mm/s and the laser power is up to 400 W. These molten pool depths are much larger than the molten pool width; i.e., the molten pool is in keyhole mode [17]. The mode of the molten pool is transformed from keyhole mode to conduction mode when the scanning speed exceeds 1200 mm/s. These molten pool depths are approximately equal to or smaller than the molten pool widths, indicating that no keyhole but only melting occurs under these processing parameters. The widths and depths of the single tracks decrease as the laser power decreases and scanning speed increases; i.e., the laser energy input reduces. From Figure 5a–c, the molten pool depth decreases from 430 μm to 25 μm when the scanning speed increases from 300 mm/s to 2100 mm/s at a fixed laser power of 500 W. As the scanning speed fixes at 300 mm/s, the depth of the molten pool decreases from 430 μm to 40 μm, as the laser power decreases from 500 W to 300 W, as shown in Figure 5a,d,g. From Figure 5, it can also be found that the SiC content is very low; this is because only 15 wt.% SiC powder is blended in the matrix and dilution occurs during the LPBF process.

As the laser energy input decreases, the molten pool shape changes from a V-shape to a U-shape; i.e., keyhole mode transfers to conduction mode. During the LPBF process, the molten pool center has the maximum temperature because of the Gauss distribution of the laser energy. Furthermore, the molten pool is very small. The molten pool temperature gradient is very large, inducing the high surface tension gradient, resultant shear stress and convective movement in the molten pool, i.e., the Marangoni effect [18]. The Marangoni effect induces a Marangoni flow, inducing the eddy currents in the molten pool, which drives the movement of SiC particles and leads to their uniform distribution in the matrix, especially under the high laser energy input.

**Figure 6:**
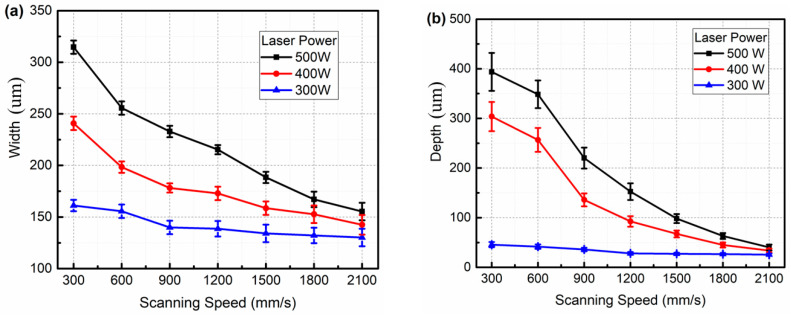
The dimensions of the single tracks with different laser power and scanning speed: (**a**) width; (**b**) depth.

The relationships between the width and depth of the single track and the processing parameters are summarized in Figure 6. As seen in Figure 6, the width and depth of the single track all decrease as the scanning speed increases at all laser powers. The width and depth of the single track are positively correlated with the molten pool volume and spreading. When the material is determined, the molten pool volume and spreading are determined by the laser energy input. If the laser energy input is large, the molten pool volume is large, the molten pool temperature is high and the molten pool lifetime is long. High temperatures lead to low viscosity of the liquid and large Marangoni flow, resulting in the molten pool spreading rapidly and sufficiently. Furthermore, the molten pool spread is also determined by the molten pool lifetime. The longer the molten pool lifetime is, the more sufficient the spreading is. Thus, a wide, deep, continuous and regular track is formed when the laser energy input is large enough [19,20]. Conversely, small laser energy input leads a small molten pool; as seen in Figure 3, Figure 4, Figure 5 and Figure 6, the temperature and lifetime of the molten pool are all low, the molten pool spreading is insufficient, resulting in the liquid phase becoming unstable and tending to break up or even forming balling. The single track splits into fragments to attain the equilibrium shape. Irregularities and discontinuities form. The morphology of the single track is deteriorated. Finally, an unstable and wrinkled surface consisting of a discontinuous length and apparent balling as well as necking is obtained when the scanning speed is 300 mm/s and the laser power is below 340 W, or when the scanning speed exceeds 1500 mm/s while laser power is kept constant at 500 W, as seen in Figure 3a,b and Figure 4e,f. 

Hence, the laser power and scanning speed, e.g., the laser energy input, play an important role in determining the geometric topography and dimensions of the single tracks together.

**Figure 7:**
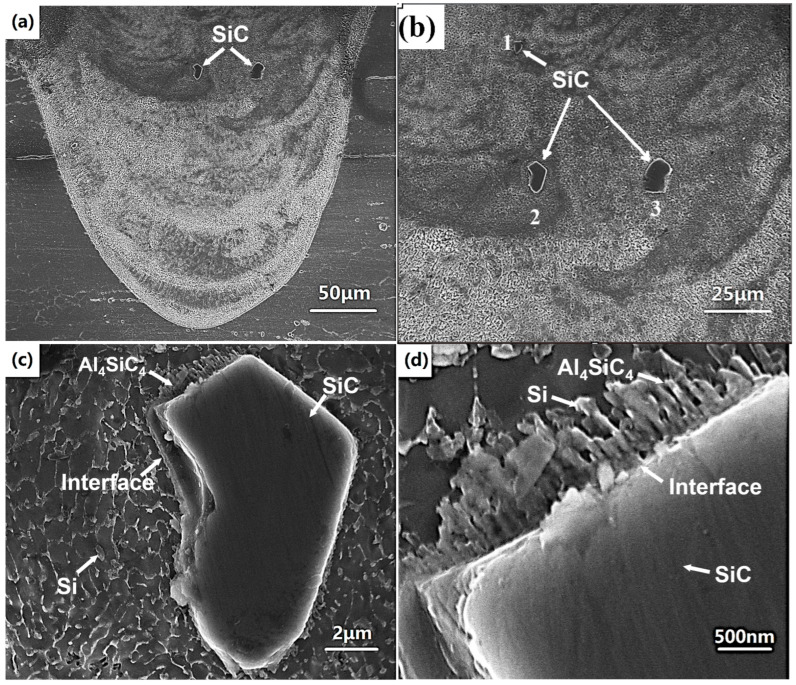
The microstructure of the single track under a laser power of 400 W and a scanning speed of 300 mm/s: (**a**) bottom of the molten pool; (**b**) upper of the molten pool; (**c**) magnification of SiC particle 2; (**d**) magnification of (**c**).

Figure 7 shows the microstructure of the molten pool of single track formed at a laser power of 400 W and scanning speed of 300 mm/s. From Figure 7a–c, it can be found that SiC particles embed in the Al matrix and the matrix consists of fine equiaxed cell structures. Furthermore, Al_4_SiC_4_ and Si faceted platelets can be found near the interface of SiC particle and Al matrix. This indicates that an interfacial chemical reaction between SiC particle and aluminum liquid occurs during the process. When the temperature exceeds 1670 K, the chemical reaction (4) occurs:

(4) $${4Al}_{\left(l\right)}+{4SiC}_{\left(S\right)}\to {{Al}_{4}{SiC}_{4}}_{\left(S\right)}+3Si$$

Al_4_SiC_4_ initially forms as faceted platelet [12]. Furthermore, some Si slices or particles form by reaction (4) near the interface, as seen in Figure 7d. Meanwhile, similar to the LPBFed AlSi10Mg, the cellular microstructure can be found in the molten pool, and eutectic Si particles are located on the boundaries of the α-Al in the form of continuous segregations because of the extreme cooling rate, as seen in Figure 7c. No pores or cracks can be found near the interface, indicating that well metallurgical bonding at the interface is achieved, as seen in Figure 7c,d. At the bottom of the molten pool, unsteady flow can be observed, which is caused by the Marangoni flow, as shown in Figure 7a.

### 3.2. Densification

**Figure 8:**
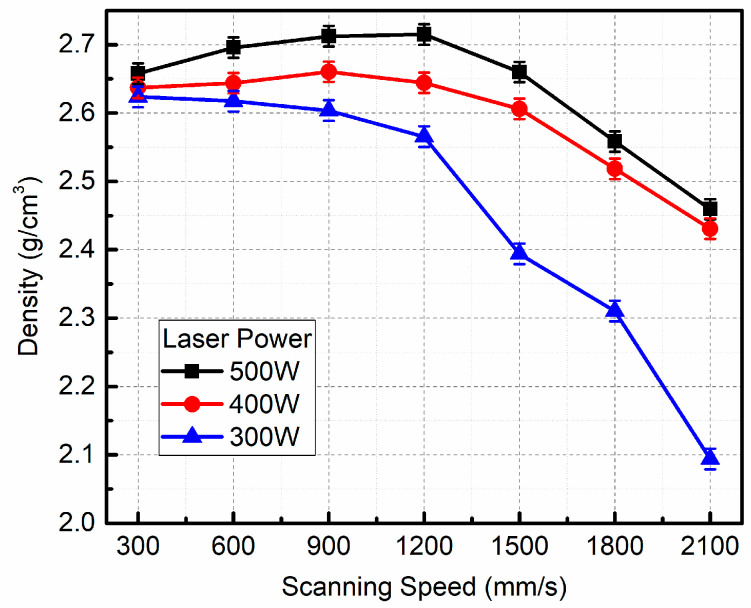
The density of LPBFed composites with different processing parameters.

Figure 8 depicts the curves of the densities of the LPBFed composites versus scanning speed under different laser powers. As the scanning speed increases, the density first increases and then decreases when the laser power is fixed at 500 W or 400 W; the density monodirectionally decreases when the laser power is fixed at 300 W. The highest density ($\rho =2.72\;g/{cm}^{3}$) is achieved when the laser power is 500 W and the scanning speed is 1200 mm/s, corresponding to 98.9% relative density $\left(\rho_{RD}\right)$, which is higher than that in our previous work using 300 mesh SiC particles [13]. When the laser power decreases, the density of the LPBFed composites decreases with fixed scanning speed. 

**Figure 9:**
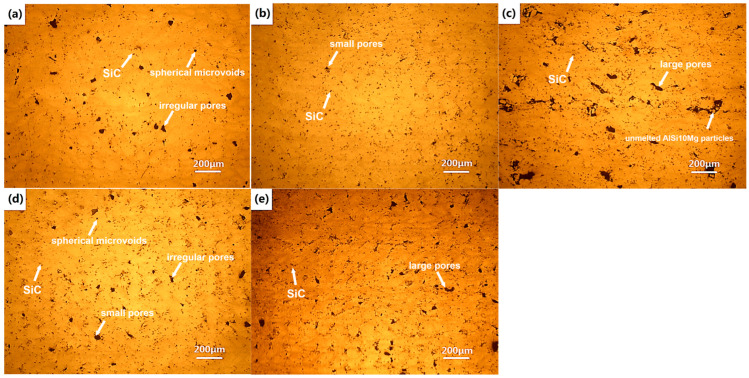
OM micrographs of LPBFed composites with different laser powers and scanning speeds: (**a**) 500 W, 300 mm/s; (**b**) 500 W, 1200 mm/s; (**c**) 500 W, 2100 mm/s; (**d**) 400 W, 1200 mm/s; and (**e**) 300 W, 1200 mm/s.

Figure 9 displays the OM micrographs of the cross-section of the LPBFed composites samples fabricated by different laser powers and scanning speeds. The distribution of SiC particles and pores, as well as the pore morphologies of the LPBFed composites samples can be seen from Figure 9. It can be seen that SiC particles distribute uniformly and different types of pores exist in the AlSi10Mg matrix.

The micrographs of LPBFed composites with a fixed laser power of 500 W and different scanning speeds of 300 mm/s, 1200 mm/s and 2100 mm/s are shown in Figure 9a–c, respectively. When the scanning speed is large, e.g., 2100 mm/s, a lot of large pores with irregular shapes and about an average size of 100 μm can be found (Figure 9c). Furthermore, unmelted AlSi10Mg powders can be observed in the matrix. Pores tend to aggregate and interconnect while unmelted powders cluster obviously, leading to the degradation of densification significantly. From Figure 4g and Figure 5c, the irregularity and discontinuity scanned track and shallow penetration depth imply that the AlSi10Mg powders cannot be completely melted during the LPBF process, inducing a number of the lack-of-fusion pores [21], which remarkably reduce the density of the LPBFed composites sample. This kind of lack-of-fusion pores are generally of irregular shape and large sizes. When the scanning speed decreases to 1200 mm/s, as shown in Figure 9b, the large irregular pores disappear but a few small pores still can be found. These processing parameters are optimal for the LPBFed composites and the highest density is achieved. However, when the scanning speed decreases to even 300 mm/s, irregular pores with an average size of about 30 μm and some spherical microvoids will be seen in the matrix, as seen in Figure 9a. Furthermore, the number of SiC particles is significantly reduced. This is because some SiC particles decompose due to the high laser energy input. When SiC particles decompose, the liquid is pushed away and the decomposition products impede the molten pool flow, which induces pores. These pores are mostly associated with SiC particles and are located at the interface of SiC particles and matrix. This kind of pores are generally in irregular shape [22]. Furthermore, the irregular pores may also be caused by the keyhole collapses during LPBF processing with high laser energy input. The small spherical microvoids may be caused by the shielding gas entrapped in the molten pool because of the extremely high laser energy input and rapid solidification of the molten pool.

Figure 9b,d,e shows micrographs of LPBFed composites samples using different laser powers and 1200 mm/s scanning speed. A small number of pores exist at a high laser power of 500 W (Figure 9b), indicating a relatively high density. If the laser power decreases, e.g., 400 W, a few observable irregular pores and microvoids will be present in the matrix, as shown in Figure 9d. Many irregular pores and some partially melted powders can be found when the laser power is 300 W (Figure 9e); i.e., the density decreases. From Figure 3a and Figure 5h, the scanned track appears unstable and wrinkled, and the molten pool is very small, which results in non-uniform layer thickness and lack-of-fusion defects. Hence, the pores are large in size, irregular in shape, and are randomly distributed, decreasing the density of the LPBFed composites.

The conclusion can be drawn that the relative density of LPBFed composites significantly depends on the laser energy input. The Marangoni driving force is a primary driving force for the densification of LPBFed composites samples, which is caused by the surface tension gradient. Since the content of oxygen is extremely low during the LPBF process and the temperature of the molten pool center is the highest, the surface tension of the liquid metal located in the molten pool center is the lowest, while the surface tension of the liquid metal located at the molten pool boundary is the highest. This induces the liquid metal to flow from center to boundary, resulting in molten pool spreading and densification occurs. The Marangoni coefficient *Ma* can represent the magnitude of the Marangoni force, which is [20]:

(5) $$M_{a}=\left|\frac{d\gamma }{dT}\right|\frac{L∆T}{\eta \alpha }\;$$

where $\frac{d\gamma }{dT}$ is the differential of surface tension with respect to temperature. L is the molten pool width. ∆T is the difference between the temperature of the molten pool center and the molten pool boundary. α is the thermal diffusivity, and η is the dynamic viscosity of the molten pool, which can be calculated by Equation (6) [23]:

(6) $$\eta =\frac{16}{15}\sqrt{\frac{m}{\kappa T}}\gamma$$

where m is the atomic mass. κ is the Boltzmann constant, and Γ is the surface tension of the molten pool, which is associated with the temperature.

Furthermore, the molten pool lifetime ∆t is also important for the densification of LPBF. Since ∆t determines the molten pool spreading time, which can be calculated according to Equation (7).

(7) $$∆t=\frac{L}{v}$$

where v is scanning speed.

With the laser power increasing or the scanning speed decreasing, e.g., the laser energy input increasing, the molten pool temperature and lifetime increase. As the molten pool temperature increases, the Marangoni coefficient (*M_a_*) increases, enhancing the movement and flowability of the liquid–solid system due to the Marangoni effect [22]. This facilitates not only the formation of stable and continuous single tracks but also promotes the flow of molten metal into pores [24], and the density of LPBFed composites increases. Furthermore, the stable and continuous track also brings a stable process, thereby improving the density of LPBFed composites. The molten pool lifetime increases, leading to sufficient spreading of the molten pool; therefore, a dense LPBFed sample is achieved. Nevertheless, the viscosity of the LPBFed composites increases greatly with the addition of SiC particles. The increased viscosity of the liquid melt results in low fluidity according to the kinetics theory. Therefore, the fully densified samples have not been obtained due to the insufficient fluidity of liquid melt. As a consequence, the optimal processing parameters for achieving the highest density LPBFed composites sample are a laser power of 500 W and a scanning speed of 1200 mm/s, with a scanning spacing of 0.12 mm and a layer thickness of 0.04 mm.

### 3.3. Microstructures

**Figure 10:**
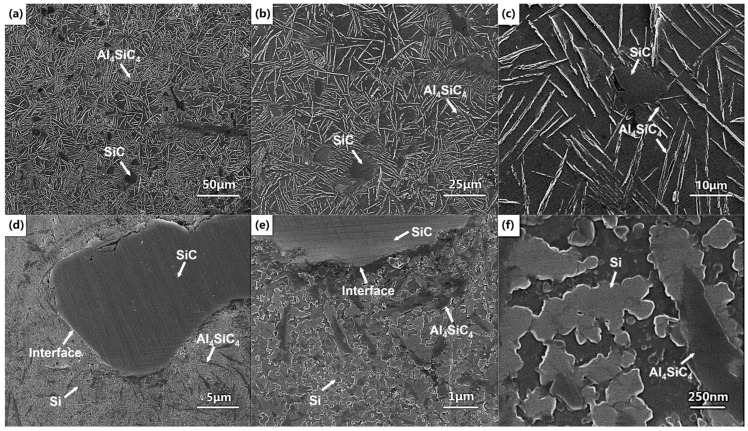
SEM images of microstructure of LPBFed composites samples under 500 W laser power and 1200 mm/s scanning speed: (**a**–**c**) polished; (**d**–**f**) etched.

The microstructure of the block sample fabricated by the optimal processing parameters is shown in Figure 10. Figure 10a–c depict the SEM photos of the polished cross-section of the LPBFed composites samples. The shape of SiC particles is similar to the shape of the starting SiC powder, exhibiting irregular morphology. Furthermore, many needle-like precipitates are observed in the matrix. The high-magnification images of needle-like precipitates are shown in Figure 10d–f. Two new phases Al_4_SiC_4_ with needle-like shapes and Si with faceted shapes are formed during the process. Similarly to the single track, the interfacial chemical reaction between SiC particle and the Al matrix also occurs in the LPBF block sample. Furthermore, compared with Figure 7 and Figure 10, it can be found that the amount of the new phases of the block sample is higher than that in the single track, indicating a stronger interfacial reaction occurring during the LPBFed block sample. This is caused by the strong thermal accumulation during the LPBFed block sample.

**Figure 11:**
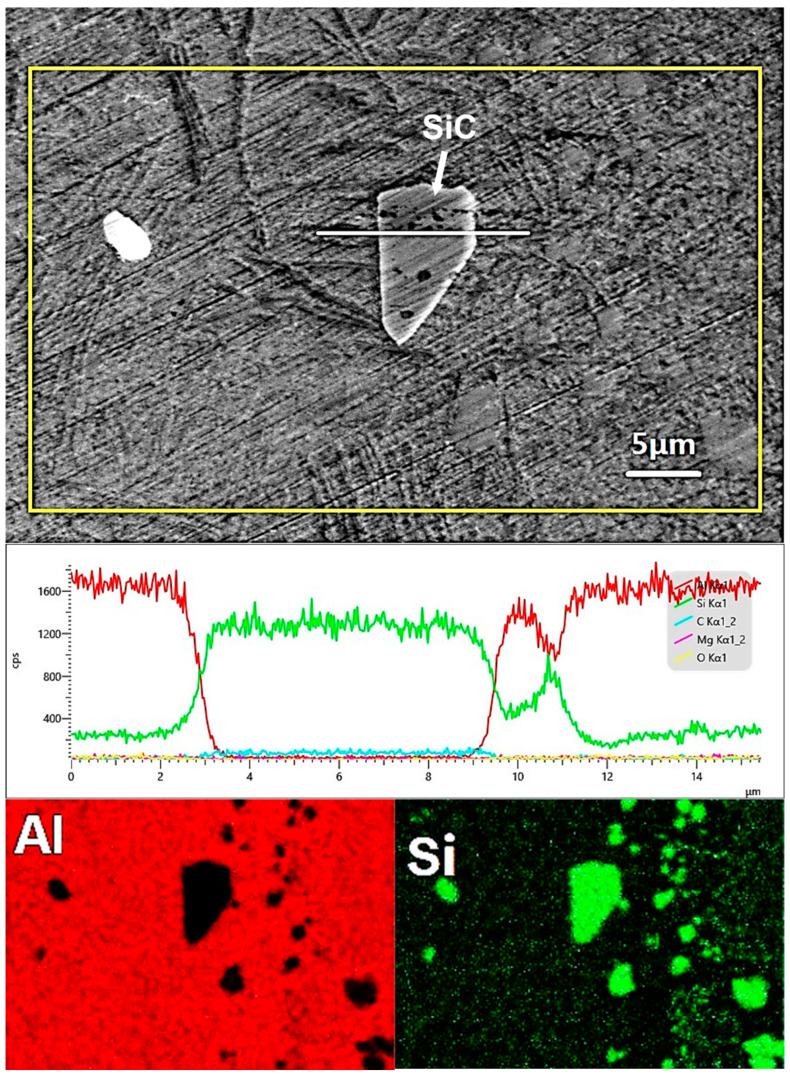
The distribution of the elements of the sample fabricated by 500 W laser power and 1200 mm/s scanning speed.

The distributions of Al and Si contents in the matrix and interface between a SiC particle and AlSi10Mg matrix are shown in Figure 11. From the line scan, a gradual change in Al and Si contents can be found at the interface, indicating an interface interlayer between the SiC particle and aluminum matrix existing. It can be found that the liquid aluminum matrix has good wettability with SiC particles from the area scan. This is caused by the high temperature during the LPBF process. Furthermore, the interfacial reaction also has a positive effect on the wettability between the SiC particles and the Al matrix. From Figure 7c, Figure 10d, and Figure 11, it is clear that the SiC particles form strong metallurgical bonding with the Al matrix both in the molten pool of single tracks and blocks. Good wettability and strong bonding are very important for improving the mechanical properties of the LPBFed composites. A fine microstructure with a homogenous distribution of alloying elements in the LPBF sample is clearly evident in the Al and Si maps. It can also be found that the faceted Si particles exist in the matrix.

### 3.4. Mechanical Properties

**Figure 12:**
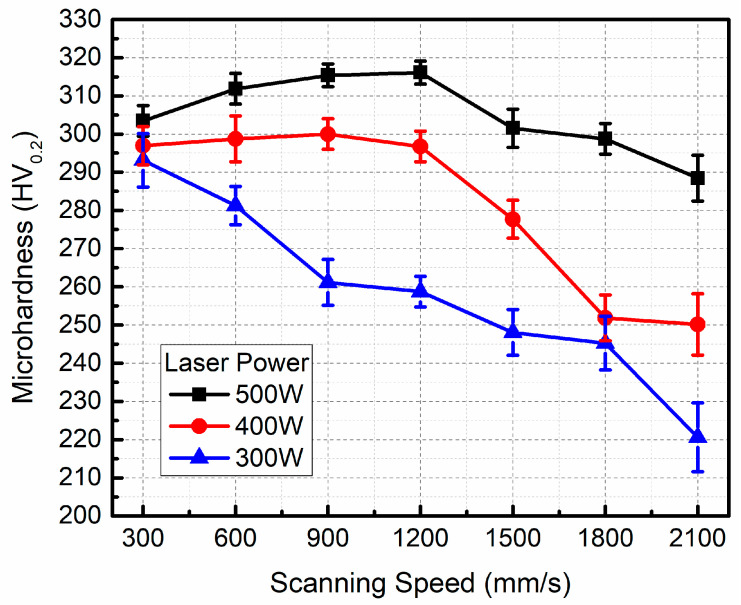
The curves of microhardness of LPBFed composites samples versus scanning speed under different laser powers.

The curves of microhardness versus the scanning speed under different laser powers are shown in Figure 12. The microhardness first increases and then decreases as the scanning speed increases at a constant laser power of 500 W or 400 W. The microhardness decreases with the increase in the scanning speed at a constant laser power of 300 W. The maximum microhardness can reach up to 316.1 HV_0.2_ when the optimal processing parameters are used, i.e., 500 W laser power and 1200 mm/s scanning speed. As the laser power decreases from 500 to 300 W, the microhardness decreases obviously. By comparing Figure 8 with Figure 12, it is obvious that the correlation between the microhardness and density is very close: the higher the density, the higher the microhardness. This is because high density means low porosity, which contributes to the microhardness. As shown in Figure 9, when the laser energy input is small, the defects in LPBFed composites are mainly large irregular lack-of-fusion pores, leading to a rapid decrease in densification and microhardness. When the laser energy input is too high, there are some small irregular pores and spherical pores in the LPBFed composites sample, and densification and microhardness all decrease lightly. Therefore, lack-of-fusion pores have a significant effect on the microhardness.

Compared with the maximum microhardness (147HV_0.2_) of LPBFed AlSi10Mg [25], the microhardness of LPBFed composites is much higher, indicating that adding SiC particles can dramatically improve the microhardness. This is because the strengthening effect of SiC particles on AlSi10Mg.

**Figure 13:**
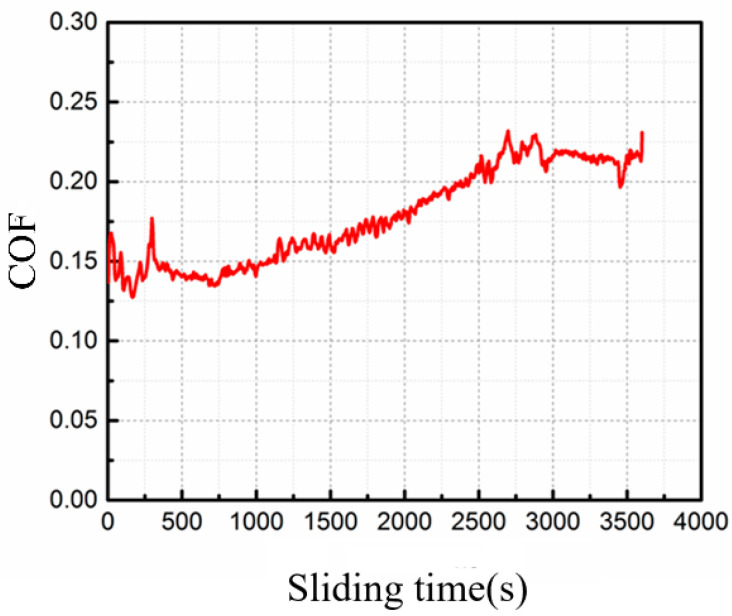
The COF of the LPBFed composites sample under 500 W laser power and 1200 mm/s scanning speed.

Figure 13 shows the coefficient of friction (COF) curve of the LPBFed composites sample fabricated by optimal processing parameters with increasing sliding time. It was found that the COF tends to rise slightly as the sliding time increases. The mean COF is 0.178. As the sliding time increases, the COF fluctuates. In the dry sliding process, a transition from mild wear to severe wear occurs. As the sliding time increases, the temperature of the sliding surface rises, leading to the matrix softening and heavy deformation. Meanwhile, the increase in COF is due to the damage of the surface layer. These induce the high COF and the wear loss of the composites. The resultant wear rate of the sample at the end of the test is 2.02 × 10^−4^ mm^3^/(N·m).

Based on Archard’s wear theory [26], the wear loss of the sample is closely related to the microhardness. Improving microhardness can enhance the wear resistance. As shown in Figure 12, the microhardness of the sample increases significantly when SiC particles are added to AlSi10Mg matrix. Therefore, wear and seizure resistance are improved. Generally, the reinforcement particles can protect the soft matrix against the deformation during sliding friction by the mechanism of resisting press-in and cutting of the sliding ceramic ball on the worn surface of the samples. Moreover, the SiC particles also enhance the thermal stability and load-bearing capacity, further improving the protection effect.

**Figure 14:**
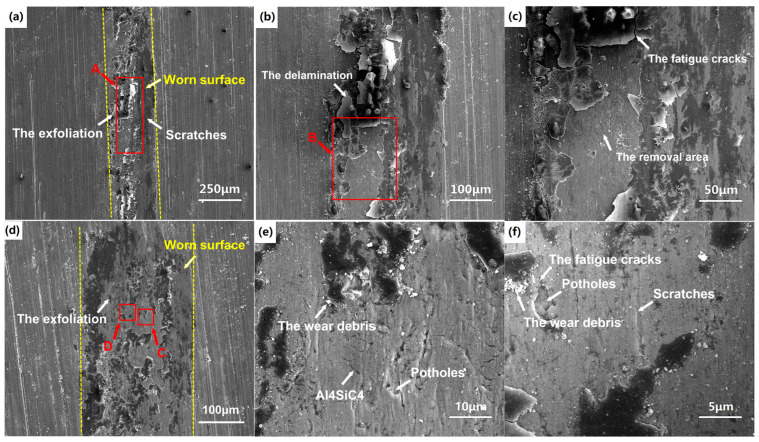
SEM images of worn surfaces of LPBFed composites sample: (**a**) surface 1; (**b**) magnification of area A in (**a**); (**c**) magnification of area B in (**b**); (**d**) surface 2; (**e**) magnification of area C in (**d**); (**f**) magnification of area D in (**d**).

The worn surfaces of the LPBFed composites sample are observed by SEM. As seen in Figure 14, the surface is characterized by scratch formation, exfoliation, and damaged regions along the sliding direction. It also shows the Al_4_SiC_4_ and the SiC particle potholes in Figure 14e,f; however, the grooves are unclear. Moreover, there is some wear debris with a granular shape on the worn surface.

Since the LPBFed composites sample has high strength, during the friction process, a strain-hardened tribolayer is formed on the worn surface as the severe plastic deformation occurs [27]. The strain-hardened tribolayer prevents the composites matrix from directly contacting the sliding ceramic ball in the initial period of friction. At this load condition, the grooves formed on the worn surface are quite shallow and narrow, indicating that adhesive wear cannot occur and the transition of the mild wear to severe wear delays. As the reciprocating motion develops, the strain-hardened tribolayer breaks up and gradually flakes off. Finally, the exfoliation forms along the friction tracks, as shown in Figure 14a,d. The SiC particles can impede the propagation of the matrix cracks, which lead to the delamination of tribolayer on the worn surface. As the cracks and exfoliation grow, delamination is removed away from the surface. Figure 14b,c clearly show the delamination wear mechanism. Moreover, some potholes can be observed due to the SiC particles pulling out, as in Figure 14e,f. The SiC particles are dislodged from the worn surface due to the propagation of the fatigue cracks. Similar observations of fatigue cracks were also reported by Tang et al. [28].

As the SiC particles are added into the matrix, the particles are well binded with the aluminum matrix during the LPBF process from Figure 10. The SiC particles act as second-body abrasives against the counterface, and the loose debris made from the SiC particles and the Al_4_SiC_4_ phases act as third-body abrasives to both the matrix and reinforcement surfaces. It can be identified that the wear scratches on the worn surface are formed due to the abrasive action of SiC particles and wear debris. In conclusion, the main wear mechanisms of the LPBFed SiCp/AlSi10Mg composites are changed to delamination and abrasive wear.

**Figure 15:**
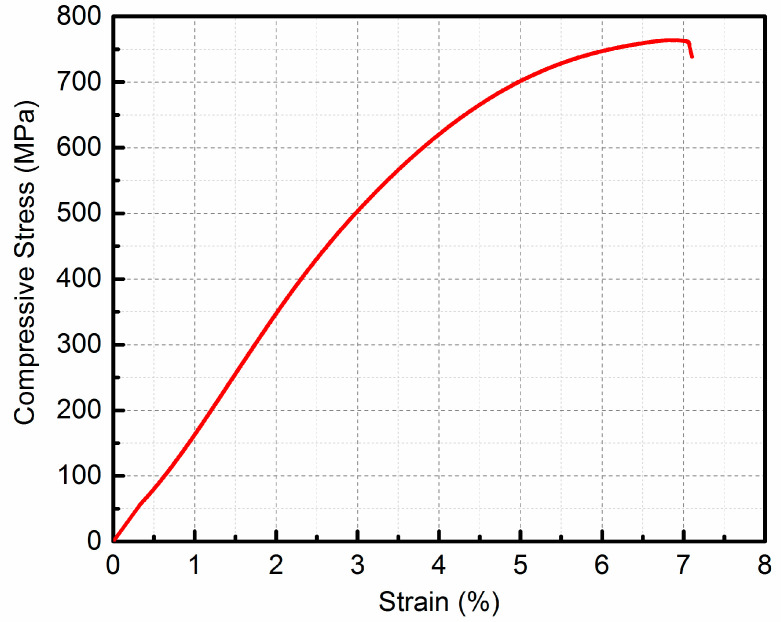
Compressive stress–strain curve of LPBFed composites sample at room temperature.

Figure 15 shows the representative compressive stress–strain curve of the LPBFed SiCp/AlSi10Mg composites at room temperature. The yield strength ($\sigma_{yc}$) and maximum compressive strength ($\sigma_{uc}$) of the composites are 566.6 MPa and 764.1 MPa, respectively. The plastic strain is not found, instead of a brittle manner. The addition of SiC particles significantly affects the mechanical behavior of the matrix alloy. Generally, the yield strength of the composites is determined by that of the matrix. Compared with the monolithic material without reinforcement particles, the stress yield is greater for the LPBFed composites. The main reason is that dislocation strengthening, fine-grained strengthening, and other mechanisms are introduced into the matrix with the addition of SiC particles. Meanwhile, the applied load is not evenly distributed between the SiC particles and the matrix due to the difference in stiffness. Hence, SiC particles bear more applied load transferring from the matrix, leading to the fracture of the matrix delays.

**Figure 16:**
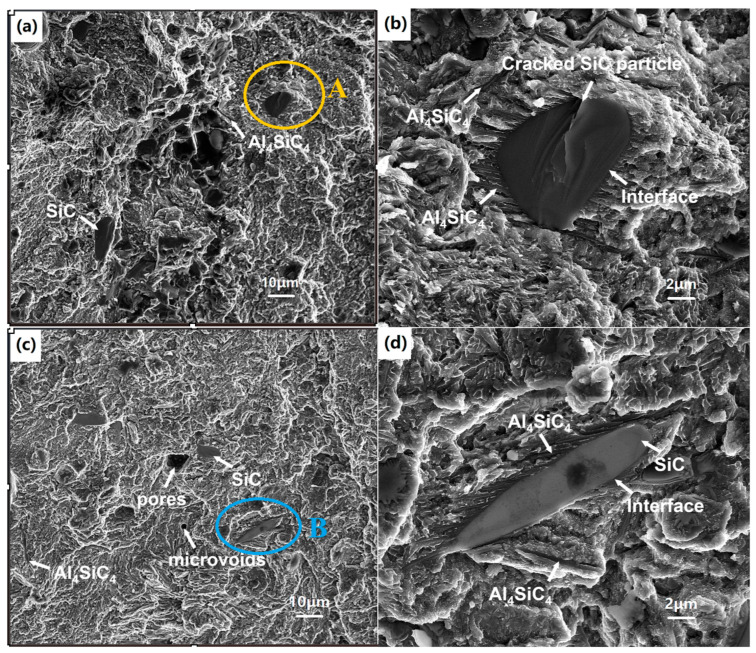
Fracture surface photos of LPBFed composites: (**a**) surface 1; (**b**) magnification of area A in (**a**); (**c**) surface 2; (**d**) magnification of area B in (**c**).

Figure 16 reveals the fracture morphology of the LPBFed composites after the compressive test. The fracture surfaces appear flat and smooth with very few dimples from Figure 16a. This indicates that the fracture is a typical brittle fracture. The cracked SiC particles which are, generally, in a smooth, flattened shape, can be found from the fracture facets, which are different from the Al matrix, as shown in Figure 16b. The SiC particles are bonded to the matrix tightly. The good interfacial bonding is due to the excellent wettability and the interfacial chemical reaction at the high temperature during the process. It can be seen from Figure 16b,d that the products of the interfacial chemical reaction, e.g., Al_4_SiC_4_, distribute near the SiC particles and in the matrix.

The cracked SiC particle can be clearly observed, as seen in Figure 16b. The surface of the SiC fracture is very smooth, with no Al matrix adhering, suggesting that the SiC particle fractured. During the compressive test, SiC particles beared the load and then broken, which have a significant effect on the ultimate compressive strength of the LPBFed composites. The initial cracks are generated at the SiC particles and then propagate into the matrix, resulting in the final fracture of the composites. Therefore, the cracking of SiC particles as a typical brittle fracture is another failure initiation mechanism in LPBFed SiCp/AlSi10Mg composites.

From Figure 9b, it can be found that a few microvoids and irregular pores exist in the microstructure. These pores have a detrimental effect on the compressive properties of the LPBFed composites. Finally, as the load increases, these pores result in the nucleation of microcracks, which promotes premature failure. It is believed that the fracture occurs either at the pores or by SiC particle cracking, rather than interface debonding.

## 4. Conclusions

The influence of the laser power and scanning speed on the characteristics of single tracks and blocks of LPBFed 15 wt.% 1200 mesh SiCp/AlSi10Mg composites was systematically investigated in this work. The microstructure and mechanical properties, as well as the wear performance of the LPBFed composite samples fabricated by the optimal processing parameters, were also tested. The main conclusions can be drawn as follows:

(1) The laser energy input has a significant effect on the surface morphology, molten pool, and dimension of the single track. As the laser energy input decreases, the width and depth of the single track decrease, the molten pool transforms from a keyhole mode to a conduction mode, and a deteriorated track with an unstable and wrinkled surface consisting of a discontinuous length and apparent balling is formed.
(2) Good metallurgical bonding between the SiC particles and Al matrix is obtained both in the molten pool of single tracks and blocks for the LPBFed SiCp/AlSi10Mg composites. During the LPBF process, the interfacial chemical reaction between SiC and the aluminum matrix occurs, forming Al_4_SiC_4_ and Si. In single tracks, the reaction products are only distributed in the interface, but they are both in the matrix and at the interface in the block samples.
(3) As the scanning speed increasing, the density increases, firstly, and then decreases when laser power is in the range of 500 W to 400 W, and decreases consistently when the laser power is 300 W. A maximum relative density of 98.9% is achieved when the laser power is 500 W and the scanning speed is 1200 mm/s.
(4) The relationship between the microhardness and processing parameters is very similar to that of the relative density versus processing parameters. The main wear mechanism is delamination wear, accompanied by abrasive wear.
(5) The yield strength and maximum compressive strength of the LPBFed composites are 566.6 MPa and 764.1 MPa, respectively. The fracture mode is mainly brittle fracture. The fracture occurs either at the pores or cracks caused by the SiC particle cracking rather than those of the interface debonding.

This work is beneficial for improving the performance of LPBFed metal matrix composite and provides a theoretical and technical foundation for LPBFed SiCp/AlSi10Mg 3D parts. Future studies could focus on exploring the influence of different SiC particle shapes and pretreatments on the properties of the LPBFed composite materials.

## Figures and Tables

**Table 1:** Processing parameters utilized in the LPBFed experiment.

No.	Processing Parameters, Unit	Value
1	Laser power *P*, W	300, 340, 370, 400, 430, 460, 500
2	Scanning speed *v*, mm/s	300, 600, 900, 1200, 1500, 1800, 2100
3	Hatching space *h*, mm	0.12
4	Layer thickness *t*, μm	40

## Data Availability

Data are contained within the article.
